# Fructose intake enhances lipoteichoic acid-mediated immune response in monocytes of healthy humans

**DOI:** 10.1016/j.redox.2025.103729

**Published:** 2025-06-12

**Authors:** Raphaela Staltner, Katja Csarmann, Amelie Geyer, Anika Nier, Anja Baumann, Ina Bergheim

**Affiliations:** Department of Nutritional Sciences, Molecular Nutritional Science, University of Vienna, Vienna, Austria

**Keywords:** Sugar, Toll like receptor 2, SP1, Gram-positive bacteria, Lipoteichoic acid, Ketohexokinase

## Abstract

Metabolic diseases like type 2 diabetes are afflicted with higher rates of infections and longer, more complicated infection course as well as higher fatality rates. The impact of nutrition and specific nutrients like free fructose herein has not yet been fully understood. Here, we performed dietary intervention studies in healthy individuals and performed *ex vivo* experiments in isolated blood immune cells to assess the effects of dietary fructose intake on Gram-positive bacterial toxin induced immune responses. Acute and extended intake of fructose but not glucose was related with an induction of *Toll like receptor* 2 mRNA expression in monocytes and enhanced the LTA-dependent release of proinflammatory cytokines from monocytes. Blocking fructose metabolism and transcription factor SP1 attenuated the fructose-related induction of *Toll like receptor* 2 mRNA expression and augmentation of proinflammatory cytokine release further suggesting that fructose-dependent metabolic alterations are critical in enhancing immune responsiveness of humans after fructose consumption.

## Highlights

• Fructose intake enhances bacterial toxin induced cytokine release in monocytes.
• Fructose but not glucose intake induces *TLR2* mRNA expression in monocytes of humans.
• Inhibition of KHK attenuated fructose-dependent enhancement of immune response.
• Fructose activates transcription factor SP1 in monocytes.

## Graphical abstract

**Figure:**
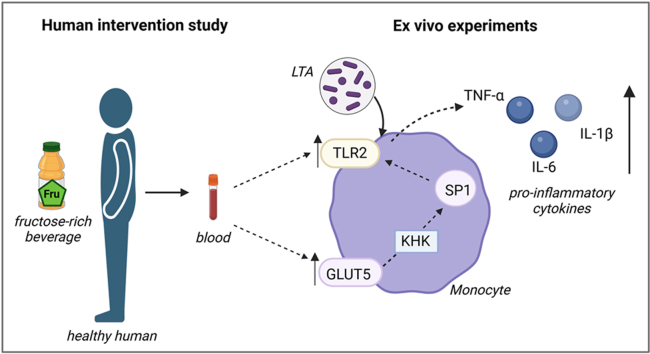

## 1 Introduction

Despite significant advance in vaccination and the development of therapeutics like antibiotics, infectious diseases caused by bacteria and viruses are still among the leading causes of deaths world-wide [1]. Indeed, it has been estimated that in 2019, 7.7 million deaths around the world were accounted to bacterial infections [2]. Taken all global deaths in consideration, this implies that 13.6 % or 1 in 8 deaths is related to bacterial infections. Studies further identified 33 bacterial pathogens across 11 infectious syndromes to be associated with bacterial infection related deaths [2]. Among these bacterial pathogens five bacteria including *Staphylococcus aureus*, *Streptococcus pneumoniae* accounted to the Gram-positive bacteria and *Escherichia coli*, *Klebsiella pneumoniae, Pseudomonas aeruginosa* accounted to the Gram-negative bacteria are responsible for more than 50 % of deaths among the investigated bacteria. In the sub-Sahara Africa super-region, higher population, poor housing, and poor sanitary infrastructure and a lack of medical care are among the key risk factors for bacterial infections and their fatality outcome [3]. In contrast, in industrialized countries it has been shown that metabolic diseases like metabolic dysfunction-associated steatotic liver disease and type 2 diabetes are afflicted with higher rates of infections, a longer and more complicated infection course and a higher fatality rate compared to healthy individuals [4,5]. Indeed, it has been discussed that the low-grade inflammation associated with metabolic diseases may gradually damage for instance liver tissue and subsequently, its immune function^4^. However, underlying mechanisms have not yet been clarified. It has also been suggested that a diet rich in certain nutrients like saturated fats and fructose may be critical in the development of metabolic diseases [6,7]. For instance, while still being discussed controversially with respect to the nutritional source of the sugar e.g., total fructose intake vs. fructose intake through sugar sweetened beverages [8], excessive fructose intake has been linked to an increased risk of metabolic dysfunction-associated steatotic liver disease (MASLD), cirrhosis, and hepatocellular carcinoma [9]. This has been accounted primarily to an increased hepatic de novo lipogenesis and reduced fatty acid oxidation, which promote liver fat accumulation and insulin resistance [10]. Also, fructose metabolism may lead to elevated uric acid levels and oxidative stress, further aggravating metabolic disturbances (for overview see Ref. [11]). Moreover, studies suggest that this “Western diet” may not only increase the odds to develop noncommunicable but also infectious diseases (for overview see Refs. [[12], [13], [14]]). Studies have also suggested that free fructose may enhance immune response to bacterial endotoxin [15]. In line with these findings, our own studies in healthy young adults suggest that an intake of high doses of free fructose is associated with increased blood levels of bacterial endotoxin and an induction of *Toll like receptor* (*TLR) 2 and* 4 mRNA expression in peripheral blood mononuclear cells (PBMCs). Interestingly, a similar alteration of *TLR2 and* 4 mRNA expression was not found after the intake of similar doses of free glucose [16]. However, whether this induction of TLRs found after the intake of elevated amounts of free fructose is primarily a result of the monosaccharide intake or the associated elevation of bacterial (endo)toxin levels in peripheral blood and if this induction of TLRs also affect immune response has not yet been clarified.

Starting from this background, the aim of the present study was to determine the effects of fructose consumption on TLR2 signaling in blood immune cells in healthy humans upon a challenge with its ligand Lipoteichoic acid (LTA) and to assess underlying molecular mechanisms.

## 2 Material and methods

### 2.1 Intervention studies

Intervention studies were carried out in accordance with the Declaration of Helsinki (revised Version 1983) and were approved by ethics committees of the University of Vienna, Vienna, Austria (reference number: 00184) and the University Hospital Jena, Jena, Germany (reference number: 4588‐11/15). All participants gave informed consent before being enrolled in the study. The human intervention studies were registered at clinical trials (NCT04788680, NCT03482284). For both studies the following exclusion criteria were defined: (1) following a special diet or (2) food malabsorption or (3) a history of diseases of the gastrointestinal tract. Furthermore, participants should be of normal-weight (BMI >18.5 kg/m^2^ or <24.9 kg/m^2^) and not drink more than moderate amounts of alcohol (<10 g/day for women; <20 g/day for men) nor smoke.

All interventions were carried out in a randomized cross-over design. Before the challenges with the different sugars dietary intake of participants enrolled in the two studies was standardized to a ‘healthy nutrition’ being in accordance with the recommendations of the German, Austrian and Swiss nutrition societies (DACH) for 2 and 4 days, respectively, as described in detail before [16,17]. In brief, after assessing dietary intake by two independent 24-h recalls, participants were asked to follow a fully balanced diet with all foods and beverages provided for 2 and 4 days, respectively, that was isocaloric to their ‘normal’ diet. In **study A**, participants were asked to provide a fasting blood sample to determine clinical parameters in serum. After the 4-day long standardization of their nutritional intake another fasting blood sample was collected and participants received either a fructose or glucose rich diet (25 % of the total daily energy intake) where both sugars were provided as jellies for another 3 days, respectively. After each intervention a further fasting blood draw was collected. For further details please also see Nier et al. [16]. PBMCs were isolated from whole blood and fructose levels in plasma were assessed as detailed below.

In **study B**, a fasting blood sample was collected before subjects were standardized in their nutritional intake to assess clinical parameters in serum. After being standardized in their nutritional intake for 2 days and an overnight fast, fasting blood was drawn. Participants were then asked to consume the respective sugar containing beverage within 1 h in combination with a light breakfast (a roll and 10 g butter). Additional blood samples were collected 120 min after the consumption of the respective beverages, which were used to isolate monocytes and to carry out stimulation experiments and to determine plasma levels of fructose as described in detail below. The study beverages consisted either of fructose (110 g) or maltodextrin (110 g) dissolved in 1 L of sparkling tap water. Doses were based on the assumption that similar amounts of sugar are found in 1 L sugar-sweetened beverages. Maltodextrin was selected over glucose to increase compliances as in pilot experiments it was found that the intake of less sweet beverages is favored by especially healthy subjects. Intake and standardization of the nutrition of the healthy subjects of both studies was assessed and calculated using the computer software EBISpro (Version 2011, Willstätt, Germany). For basic characteristics of the study subjects of **study A and B** see Table S1 and S2. For results of 24-h recalls and standardization of nutrition of **study B** see Table S3.

### 2.2 Isolation of PBMCs, monocytes and B- as well as T-cells

PBMCs, monocytes and B- as well as T-cells, respectively, were either isolated from whole blood samples obtained from participants of **study A and B** or from healthy donors, and buffy coats. Buffy coats for the isolation of cells were obtained from the ‘Austrian Rotes Kreuz’. In addition, after obtaining written informed consent and an overnight fast, blood was drawn from healthy, normal weight donors which was approved by the ethics committees of the University of Vienna, Vienna, Austria (reference number: 00586). Cells were isolated by density gradient centrifugation using pluriSelect system following the instructions of the manufacturer (pluriSelect Life Science UG & Co. KG, Leipzig, Germany). In brief, blood or buffy coats were layered onto monocytes density gradient media (1.069 g/ml) in pluriSelect separation tubes and centrifuged at room temperature for 15 min for isolating monocytes. PBMCs were isolated by layering blood onto Pancoll solution (1.077 g/ml) and centrifugation for 30 min at room temperature. B- and T-cells were obtained in a second step by layering PBMC fraction onto an iso-osmotic Percoll solution (46 %) and centrifugation for 30 min at room temperature. Cells were collected and used for further experiments (see below).

### 2.3 Stimulation of monocytes obtained after an oral challenge with fructose or maltodextrin with a TLR2 ligand

After consumption of the test beverage in **study B**, isolated monocytes were seeded in 24-well plates in RPMI medium (RPMI-1640-Medium without glucose, with l-glutamine and sodium bicarbonate; Sigma Aldrich, Darmstadt, Germany) containing 10 % fetal bovine serum (Pan-Biotech GmbH, Aidenbach, Germay) and 100 μg/ml streptomycin and 100 U/ml penicillin (Pan-Biotech GmbH, Aidenbach, Germany) in a 5 % carbon dioxide atmosphere. Monocytes were then challenged with or without lipoteichoic acid (LTA, 10 μg/ml, derived from *Staphylococcus aureus*, Sigma-Aldrich) for additional 2 h. LTA is a component of the wall of Gram-positive bacteria including *Staphylococcus aureus* and acts as a pathogen-associated molecular pattern (PAMP) binding to TLR2 and leading to an activation of dependent signaling cascades [18]. Cells and supernatant of cells were collected for measuring mRNA expression and protein concentration of cytokines (see below).

### 2.4 Experiments in PBMCs, monocytes as well as B- and T-cells isolated from buffy coats and healthy donors

Isolated PBMCs, monocytes as well as B- and T-cells were seeded in 24 well plates by using RPMI medium (RPMI-1640-Medium without glucose, with l-glutamine and sodium bicarbonate; Sigma Aldrich, Darmstadt, Germany) containing 10 % fetal bovine serum (Pan-Biotech GmbH, Aidenbach, Germay) and 100 μg/ml streptomycin and 100 U/ml penicillin (Pan-Biotech GmbH, Aidenbach, Germany) in a 5 % carbon dioxide atmosphere. In a **first set of experiments,** PBMCs were treated with fructose (50 μM) for 2, 6 and 24 h while monocytes as well as B- and T-cells were treated with fructose (50 μM) for 2, 6 or 12 h. For assessing the mRNA expression of proinflammatory cytokines, monocytes were treated with fructose (50 μM) for 12 h followed by stimulation with LTA (10 μg/ml) for additional 2 h in a **second set of experiments.** Furthermore, in a **third set of experiments,** monocytes treated with fructose (50 μM) were concomitantly treated with the SP1 inhibitor MIT (mithramycin, 10 nM) or the KHK inhibitor (KHK inhibitor 1, N8-(Cyclopropylmethyl)-N4-(2(methylthio)phenyl)-2-(1-piperazinyl)-pyrimidol[5,4-*d*]pyrimidine-4,8-diamine (1 μM)) for 12 h. In a **fourth set of experiments** monocytes were treated with fructose (50 μM) and the KHK inhibitor (1 μM) for 12 h and further incubated with LTA (10 μg/ml) for 2 h. Supernatant and cells were collected after the incubation, respectively for further analysis.

### 2.5 Caco-2 *trans*-well model

The study design is summarized in Fig. 3a. Differentiated Caco-2 cells (ACC 169, DSMZ, Braunschweig, Germany) were grown in *trans*-wells by using DMEM medium containing 10 % fetal bovine serum (Pan-Biotech GmbH, Aidenbach, Germay) and 100 μg/ml streptomycin and 100 U/ml penicillin (Pan-Biotech GmbH, Aidenbach, Germany) in a 5 % carbon dioxide atmosphere. Monocytes were seeded in the basolateral compartment of the *trans*-well system. In a **first set of experiments,** after reaching confluency, Caco-2 cells were treated apical with fructose (10 mM) for 14 h and collected for further assessment of GLUT5 protein concentration. Furthermore, in a **second set of experiments,** after Caco-2 wells were exposed to fructose (10 mM) for 14 h, human monocytes in the basolateral compartment were then stimulated with LTA (10 μg/ml) for 2 h. Caco-2 cells and monocytes were lysed in TRItidy G (peqGOLD Trifast, Peqlab, Germany) for subsequent analysis.

### 2.6 Fructose assay

Fructose concentration was measured in plasma of human participants of **study A** and **study B** as well as in the basolateral compartment of the Caco-2 *trans*-well using a commercially available fructose assay kit (Abnova GmbH, Heidelberg, Germany).

### 2.7 ATP assay

Monocytes isolated from buffy coats were lysed in a buffer consisting of 50 mM Tris HCl, 150 mM NaCl, 2 mM EDTA, 1 % Nonidet P40. ATP levels were measured using a commercially available cellTiter-Glo Luminescent Cell Viability Assay (Promega GmbH, Walldorf, Germany) following the instructions of the manufacturer.

### 2.8 RNA extraction and real-time PCR

RNA was extracted from monocytes, B- and T-cells and PBMCs using TRItidy G (peqGOLD Trifast, Peqlab, Germany) and reverse transcribed using a cDNA synthesis kit (Promega, Walldorf, Germany) following the instructions of the manufacturer. Primers used to determine *GLUT5*, cytokines (*TNFα, IL-1β, IL-6*), Toll like receptors (*TLR1, 2, 6*), ketohexokinase (*KHK*) and *SP1* mRNA expression were designed using the software Primer 3. To determine the amount of target genes, normalized on endogenous reference (*18S*) and relative to a calibrator (2−ΔΔCt), the comparative CT method was used as detailed previously. Primer sequences are shown in Table S4.

### 2.9 Enzyme-linked Immunosorbent assay (ELISA)

Protein concentrations of IL-1β, IL-6, TNF-α, and soluble TLR2 were analyzed in cell culture supernatant of the monocyte *in vitro* experiment as well as of the human intervention **study B** using commercially available ELISA kits (Bio-Techne Corp., Minneapolis, MN, USA) according to the manufacturer.

### 2.10 Western blot analysis

To obtain total protein, Caco-2 cells and monocytes were lysed in TRItidy G (ITW Reagents, S.R.L, Milano, Italy) according to the instructions of the manufacturer. Protein was then separated in a gel electrophoresis and transferred onto a PVDF membrane. Membranes were then incubated with anti-GLUT5, anti-SP1 (Santa Cruz Biotechnology, Dallas, TX, USA), or anti-β-actin (Santa Cruz Biotechnology, Dallas, TX, USA) at 4 °C overnight, and subsequently incubated with a secondary antibody (anti-mouse or anti-rabbit, respectively Cell Signaling Technology, Danvers, MA, USA). Band intensity was analyzed with Super Signal West Dura Kit (Thermo Fisher Scientific, Waltham, MA, USA) and densities of the bands were analyzed with ChemiDoc XRS system as previously described [19].

### 2.11 Statistical analysis

Data are presented as mean ± SEM. Grubb's test was performed before statistical analysis to identify outliers. Normality of distribution of data was analyzed using Shapiro-Wilk normality test. Differences between two paired groups were assessed using a paired T-Test for each time point, respectively. Data were log-transformed if not normally distributed. All data were analyzed with GraphPad Prism software (GraphPad Prism Software Inc., USA). A p-value ≤0.05 was considered significant.

## 3 Results

### 3.1 Effect of fructose on *TLR2* and *GLUT5* expression in PBMCs, monocytes, B- and T-cells

To determine if the induction of *TLR2* mRNA expression in PBMCs found previously in healthy subjects consuming a fructose enriched diet [16] was a result of the sugar itself and in which subfraction of PBMCs *TLR2* mRNA expression was induced, we stimulated monocytes, B- and T cells isolated from buffy coats with fructose (50 μM) for 2, 6, and 12 h (for experimental set-up see Fig. 1a). Expression of *TLR2* mRNA was significantly induced in monocytes exposed to fructose after 6 and 12 h while no changes in *TLR2* mRNA were found after 2 h when compared to naïve cells (Fig. 1b). Similar effects were not found when cells were exposed to a glucose enriched cell culture media (see Fig. S1). Also, expressions of *TLR1* and 6 mRNA were not altered in monocytes exposed to fructose at any of these timepoints (see Table 1). Furthermore, *TLR2* mRNA expression was below the level of detection in B- and T-cells regardless of additional treatments (Fig. 1b).

**Fig. 1:**
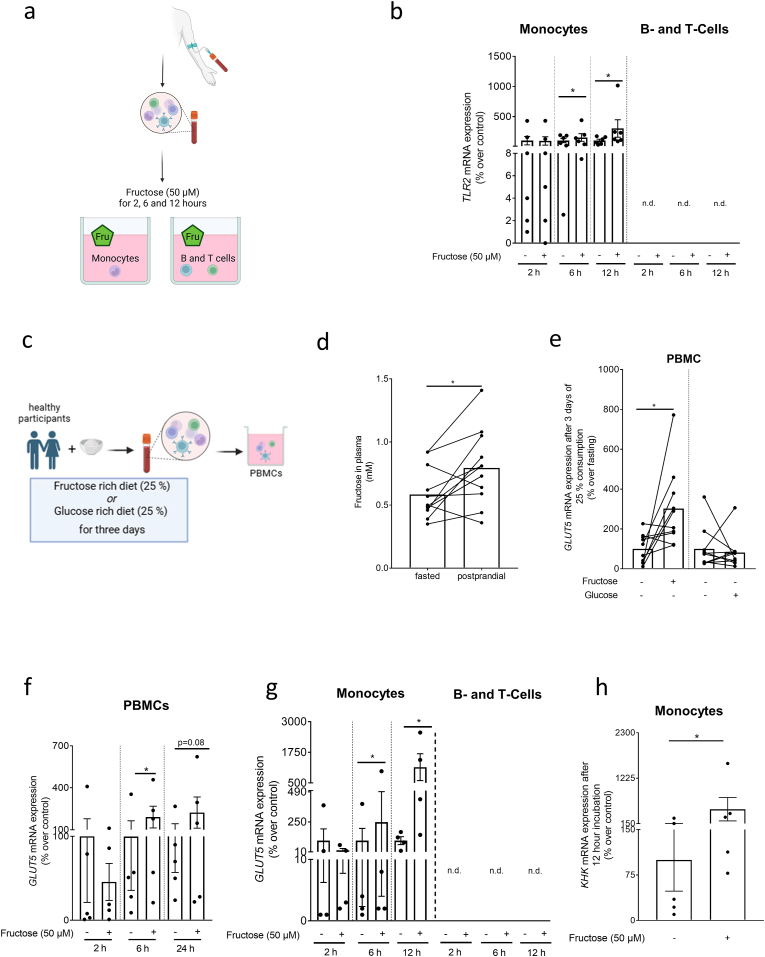
**Expression of *TLR2, GLUT5,* and *KHK* mRNA in PBMCs, monocytes, and B- as well as T-cells isolated from healthy donors or healthy humans after the intake of fructose**. (a) Schematic drawing of experimental set-up of experiments shown in (b) and (f, g, h). (b) *TLR2* mRNA expression in monocytes and B- and T-lymphocytes isolated from buffy coats and *ex vivo* challenged with fructose (50 μM) for 2, 6 and 12 h. (c) Study design of the human intervention study (study A) with an acute intake of fructose (40 g) and a fructose rich diet for 3 days (25 % E of total caloric intake). (d) Fructose levels in plasma before and 3 h after a light breakfast containing 40 g fructose as well as (e) *GLUT5* mRNA expression in isolated PBMCs after three days of a fructose or glucose rich diet (25 % E of total caloric intake) of healthy participants. (f) *GLUT5* mRNA expression in PBMCs from healthy human donors challenged *ex vivo* with fructose (50 μM) for 2, 6 and 24 h. (g) *GLUT5* mRNA expression in monocytes and B- and T-cells of healthy human donor's *ex vivo* challenged with fructose (50 μM) for 2, 6 and 12 h. (h) *KHK* mRNA expression in monocytes isolated from buffy coats and challenged with fructose (50 μM) for 12 h *∗p≤0.05*. Fig. 1 (a); n = 6; Fig. (d, e): n = 10; Fig. (f): n = 5; Fig. (g): n = 4; Figure (h): n = 5. Fig. 1 (a) and (c) were created with biorender.com. TLR2: Toll like receptor 2; GLUT5: glucose transporter 5; PBMC: peripheral blood mononuclear cells: KHK: ketohexokinase.

**Table 1:** Expression of *TLR1* and *6* in human monocytes isolated from buffy coats and incubated ±50 μM fructose for 2, 6 or 12 h.

Parameter	2 h		6 h		12 h	
	C	Fructose (50 μM)	C	Fructose (50 μM)	C	Fructose (50 μM)
*TLR1* mRNA expression**^#^**	100	114.7 ± 35.1	100	113.8 ± 28.9	100	64.9 ± 11.9
*TLR6* mRNA expression**^#^**	100	81.0 ± 31.2	100	89.0 ± 21.1	100	90.0 ± 29.7

Values are means ± standard error of means. # % over control; n = 6–9; C: untreated cells, control; TLR: Toll like receptor.

To determine if the fructose-dependent induction of *TLR2* was related to changes in fructose uptake and metabolism in PBMCs and monocytes, respectively, samples from healthy subjects that were asked to consume fructose (25 E%) or glucose (25 E%) in a cross-over design study were analyzed (study design Fig. 1c and Table S1 as well as Nier et al. [16]). Three hours after the intake of fructose a significant post-prandial increase in serum fructose levels was observed (Fig. 1d). Moreover, compared to baseline, glucose transporter 5 (*GLUT5)* mRNA expression in PBMCs was induced by ∼3-fold after subjects had consumed the fructose enriched diet for 3 days (Fig. 1e). A similar increase in *GLUT5* mRNA expression in PBMCs was not found after the intake of the glucose enriched diet in which all other foods and beverages were similar to those consumed when subjects were exposed to fructose (Fig. 1d and e).

Furthermore, challenging PBMCs *ex vivo* with fructose for 2, 6, and 24 h resulted in a significant and by trend higher induction of *GLUT5* mRNA expression after 6 h (p ≤ 0.05) and 24 h (p = 0.08) compared to naïve controls (Fig. 1f). To further determine in which cell population *GLUT5* mRNA expression was altered, we next challenged monocytes and B- as well as T-cells with fructose for 2, 6, and 12 h. *GLUT5* mRNA expression was only detectable in monocytes and was significantly induced after 6 and 12 h when compared to naïve controls (Fig. 1g). Moreover, mRNA expression of ketohexokinase (*KHK)* being the pacemaker enzyme of fructose metabolism [20], was also significantly induced by ∼5-fold in monocytes exposed to fructose for 12 h (Fig. 1h).

### 3.2 Effect of fructose on LTA-dependent stimulation of monocytes

**Fig. 2:**
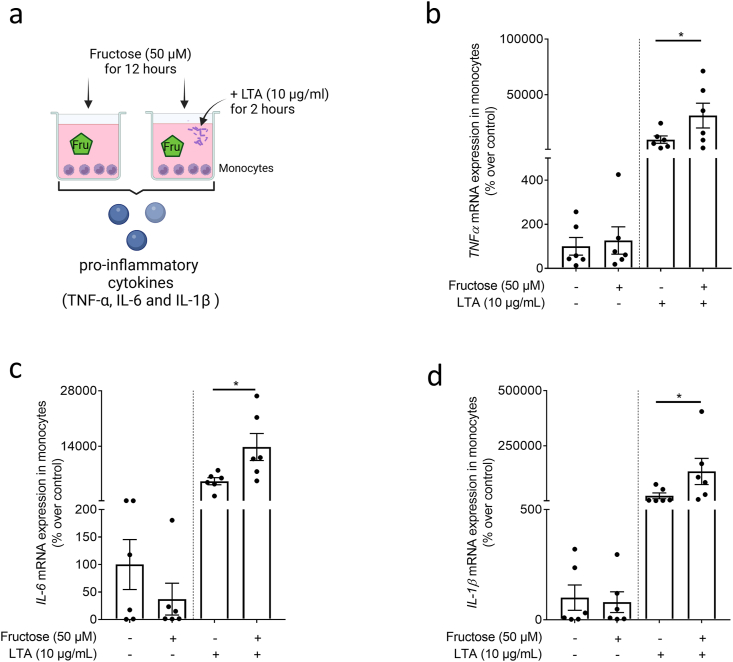
**Effect of fructose on the LTA-dependent induction of proinflammatory cytokine expression in monocytes obtained from healthy donors**. (a) Graphical illustration of experimental set-up. (b) *TNFα*, (c) *IL-6* and (d) *IL-1β* mRNA expression in monocytes pre-incubated for 12 h with 50 μM fructose and challenged with 10 μg/ml LTA for 2 h n = 6. *∗p≤0.05*. Fig. 2 (a) was created with biorender.com. LTA: lipoteichoic acid; TNFα: tumor necrosis factor; IL: interleukin.

**Fig. 3:**
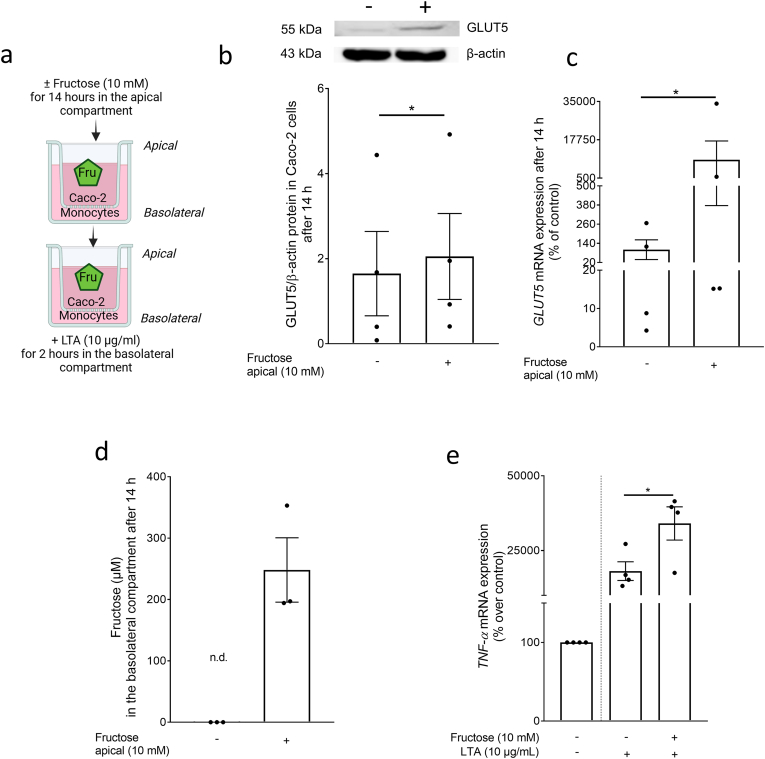
**Effect of fructose on fructose transport and the LTA-dependent induction of *TNFα* mRNA expression in a co-culture model of differentiated Caco-2 cells and human primary monocytes**. (a) Experimental set-up. (b) GLUT5 protein concentration in Caco-2 cells and (c) *GLUT5* mRNA expression in monocytes 14 h after challenging Caco-2 cells with 0–10 mM fructose (d) fructose concentration in the basolateral compartment as well as (e) *TNFα* mRNA expression in monocytes after the 14 h challenge of Caco-2 cells with fructose (10 mM) and 2 h incubation of monocytes with LTA (10 μg/ml). n = 3–4. *∗p≤0.05.*Fig. 3 (a) was created with biorender.com. GLUT5: glucose transporter 5; LTA: lipoteichoic acid; TNFα: tumor necrosis factor.

To determine if the exposure to fructose also affects the immune response of monocytes to lipoteichoic acid (LTA), the latter being derived from the walls of *Staphylococcus aureus* and has been shown before to activate immune cells through TLR2-dependent signaling cascades [21], monocytes pre-exposed to a fructose enriched cell culture media were challenged with 10 μg/ml LTA (Fig. 2a for experimental set-up). Expressions of *tumor necrosis factor alpha (TNFα), interleukin-6 (IL-6), and interleukin-1β (IL-1β)* mRNA were significantly enhanced by ∼3.2-, ∼2.7-, and ∼5.8-fold in LTA-treated cells pre-exposed to fructose compared to cells only treated with LTA. Exposure to fructose alone had no effects on the mRNA expression of the three cytokines when compared to naïve cells (Fig. 2b–d). Moreover, when exposing differentiated Caco-2 cells grown in a *trans*-well system for 14 h to fructose (10 mM) on the apical side of the *trans*-well system, GLUT5 protein levels in Caco-2 cells were significant increased by ∼1.2-fold while in monocytes cultivated in the basolateral compartment of the *trans*-well system, mRNA expression of *GLUT5* was induced by ∼8-fold compared to controls (Fig. 3, b + c). Fructose levels in the basolateral compartment of the co-culture model increased from 0 μM to ∼250 μM (∼2.5 % of the fructose concentration added to the apical compartment, Fig. 3d). The addition of LTA for 2 h to the basolateral compartment of *trans*-wells exposed to fructose resulted in a significantly higher expression of *TNFα* mRNA (+∼1.9-fold) in monocytes when compared to monocytes cultivated underneath Caco-2 cells treated with plain cell culture media (Fig. 3e).

### 3.3 Effect of fructose and maltodextrin intake in healthy human subjects on the LTA-dependent induction of proinflammatory cytokines in monocytes

**Fig. 4:**
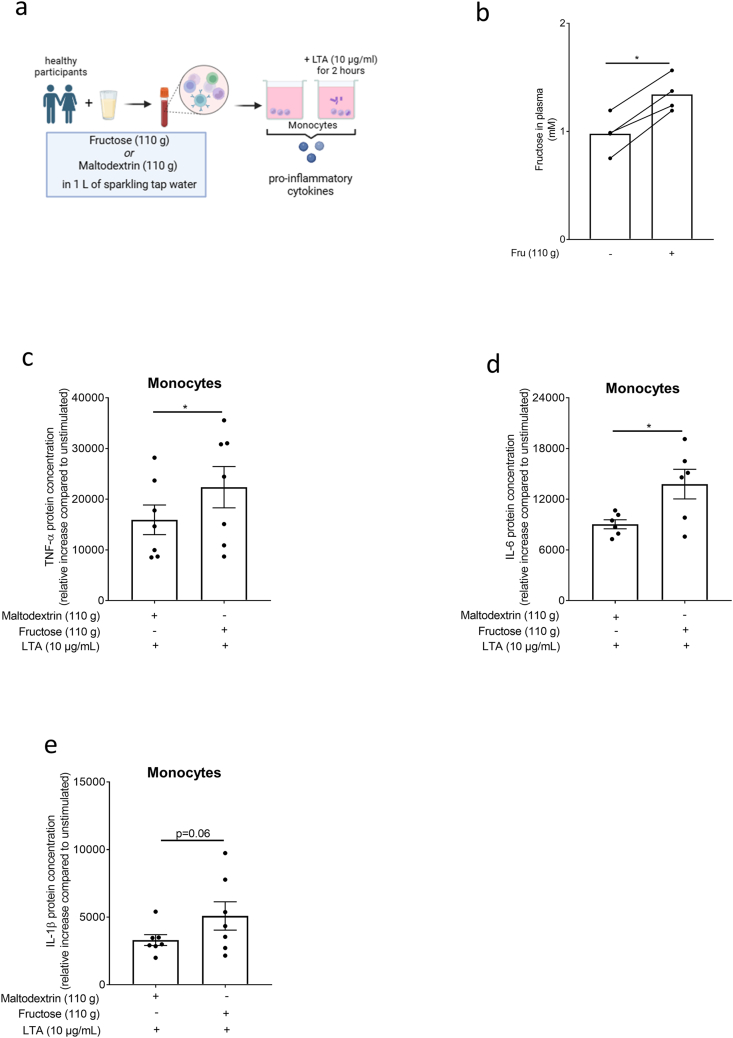
**Effect of an acute intake of an isocaloric fructose- or maltodextrin-rich beverage on fructose levels in blood and LTA-induced cytokine release in monocytes**. (a) Study design of the human intervention study (study B) and *ex vivo* experiments. (b) Plasma fructose levels 2 h after the intake of 110 g fructose. Relative increase (compared to unstimulated) of (c) TNFα, (d) IL-6, and (e*)* IL-1β protein concentration in monocytes isolated after the intake of maltodextrin or fructose challenged *ex vivo* with and without 10 μg/ml LTA for 2 h n = 4–7. *∗p≤0.05.*Fig. 4 (a) was created with biorender.com.; LTA: lipoteichoic acid; TNFα: tumor necrosis factor; IL: interleukin.

To determine if fructose also alters immune responsiveness of monocytes after an acute ingestion of fructose in humans, healthy subjects were asked to either consume 1 L of a fructose or a maltodextrin sweetened beverage containing 110 g of either sugar after being standardized in their dietary intake for 2 days (for study design and baseline characteristics see Fig. 4a and Supplemental Table S2). The intake of the fructose enriched beverage was again related with a significant increase in plasma fructose levels (Fig. 4b). Moreover, upon stimulation with LTA, the relative release (compared to unstimulated) of TNFα and IL-6 proteins from monocytes isolated after fructose ingestion was significantly higher by ∼1.4- and ∼1.5-fold, respectively, compared to that found after subjects consumed maltodextrin. After the fructose intake, IL-1β protein levels in the cell culture media of monocytes challenged with LTA were ∼1.5-fold higher (p = 0.06) than that detected after the intake of maltodextrin (Fig. 4c–e).

### 3.4 Effect of a SP1 inhibitor mithramycin and a ketohexokinase inhibitor on the fructose- and LTA-related induction of TLR2 and proinflammatory cytokines in monocytes

**Fig. 5:**
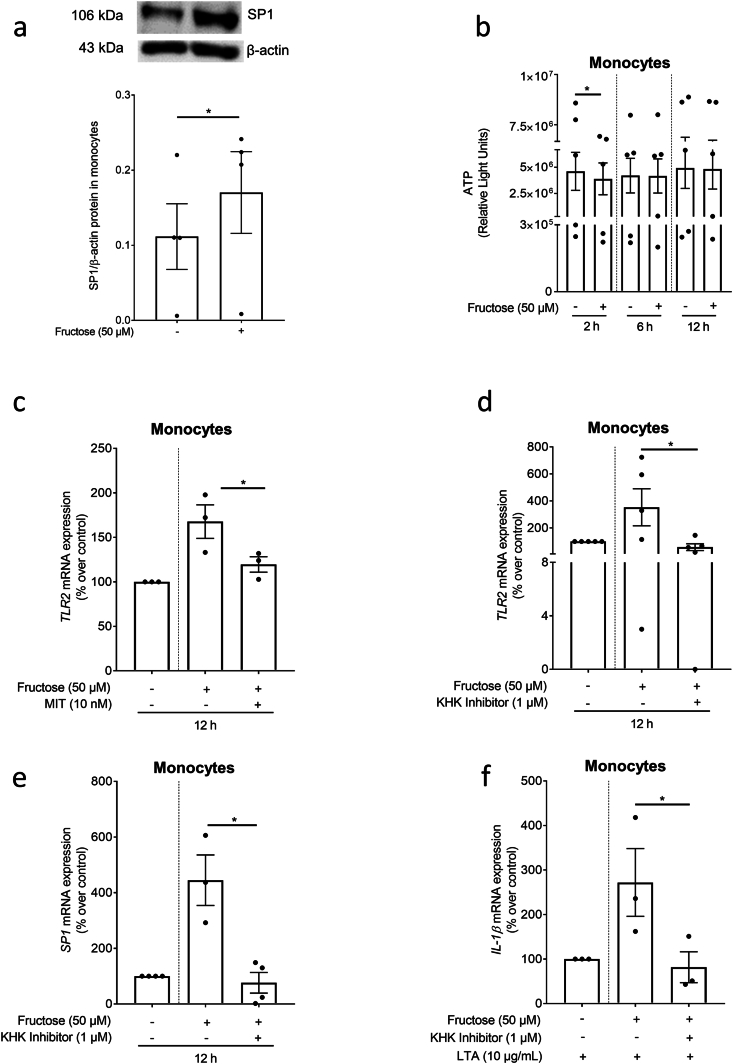
**Effect of fructose on SP1 protein and ATP levels in primary monocytes and on SP1 and KHK-1-dependent regulation of *TLR2* expression in primary monocytes**. Effect of the incubation of monocytes with fructose (0–50 μM) for 12 h on (a) SP1 protein concentration. (b) ATP units in monocytes after 2, 6 and 12 h of incubation with fructose. *TLR2* mRNA expression in monocytes incubated with fructose (c) ± MIT (10 nM) and (d) ± KHK inhibitor 1. (e) *SP1* mRNA expression of monocytes incubated with fructose±KHK inhibitor 1 (1 μM) and (f) IL-*1β* mRNA expression after additional stimulation with LTA (10 μg/ml) for 2 h n = 3–5. *∗p≤0.05*. MIT: mithramycin; SP1: transcription factor 1; TLR2: Toll like receptor 2; KHK: ketohexokinase.

As studies suggest that the transcription factor SP1 may be critical in the regulation of TLR2 and fructose has been suggested to alter SP1 activity [22], we next determined if SP1 is critical in the fructose-dependent induction of TLR2 in monocytes (Fig. 5). SP1 protein levels were significantly higher in cell homogenates of fructose-challenged monocytes compared to control cells (Fig. 5a). The induction of SP1 was related with a significant decrease of ATP levels in cells challenged with fructose for 2 h which was no longer detectable 6 and 12 h after the fructose challenge was initiated (Fig. 5b). Furthermore, pre-treating isolated monocytes with a SP1 inhibitor (mithramycin) and a KHK inhibitor (KHK inhibitor 1), respectively, blunted the fructose-dependent induction of *TLR2* mRNA expression almost completely to the level of controls (Fig. 5 c + d). Moreover, *SP1* mRNA expression was significantly lower in fructose-treated monocytes concomitantly treated with KHK inhibitor 1 compared to fructose + vehicle-treated cells (Fig. 5e). After incubating fructose-treated monocytes with KHK inhibitor 1 for 12 h and a challenge with LTA for further 2 h the mRNA expression of *IL-1β* was significantly lower compared to monocytes without the inhibitor (Fig. 5f).

## 4 Discussion

Studies suggested that especially in industrialized countries nutritional intake and dietary pattern may not only be related to the development of metabolic diseases but may also increase the susceptibility to (bacterial) infectious diseases [23]. While intense research efforts have been attributed to unravel molecular mechanisms underlying bacterial infections and to develop new treatment measures besides antibiotics, the understanding of the role of nutrition and specific nutrients in the development and progression of bacterial infections is still limited. Employing short-term human intervention studies and *ex vivo* stimulation experiments in primary human immune cells we here report that the intake of free fructose is related with an induction of *TLR2* expression in monocytes and subsequently an increased responsiveness of these cells to the Gram-positive bacteria derived toxin LTA. The amounts of fructose employed in the present acute and short-term intervention study may seem rather high (44–110 g, for comparison, 1 L of commercially caffein-free soft drink on average contains 90 g of sucrose (sucrose in Europe) translating into 45 g of fructose). Studies conducted in industrialized countries suggest that especially in adolescents and young adults these amounts of fructose can be reached on a daily basis when sugar sweetened beverages (e.g., soft drinks and yoghurt drinks) and sweets (e.g., chocolates, chewy sweets, and confectioneries) are included in the diet [24]. In the present study we found that an acute intake of this amount of free fructose resulted in a significant increase of fructose levels in peripheral blood within 2 h after ingestion being related to a significant increase in *GLUT5 and KHK* mRNA expression in monocytes. Effects alike were not found in B- and T-cells. Moreover, we found a marked induction of *GLUT5* mRNA expression in primary human monocytes cultivated below Caco-2 cells grown on *trans*-well inserts upon exposure of Caco-2 cells to moderate amounts of fructose. Interestingly, while Caco-2 cells were exposed to 10 mM fructose apical, after 14 h, fructose concentration in the basolateral compartment was only ∼250 μM suggesting that either Caco-2 cells and/or monocytes rather efficiently metabolize fructose. It has been shown before by others that the oral ingestion of fructose is related with increased levels of the monosaccharide in peripheral blood similar to those found in the present study [[25], [26], [27]]. Also, others reported before that in enterocytes *GLUT5* mRNA expression is rather rapidly induced upon exposure to fructose and that when challenged with ‘normal’ doses of the monosaccharide the greater proportion of it is metabolized into glucose and triglycerides, while only limited amounts are being released as fructose into the circulation at the basolateral side of the cells [28,29]. Results of *in vitro* studies suggest that GLUT5 almost exclusively functions as d-fructose [30] transporter. In line with our findings, results of others also suggested before, that monocytes metabolize fructose [31].

Somewhat contrasting the findings of the present study, it has been reported by others that a chronic exposure to high amounts of fructose may alter B- and T-cell development and function in rodents [32,33]. Also, *in vitro* studies suggest that B-cells show mitochondrial oxidation of fructose when the sugar is administrated alone [34] whereas studies in mice suggest that fructose-related effects on T-cells e.g., in priming antitumor CD8^+^-T-cells or modulating T-cells population, are related to indirect effects involving other cells or intestinal microbiota [35,36]. In line with our findings, Jones et al., also reported no direct effects of fructose on cell proliferation when challenging CD4^+^- and CD8^+^-T-cells [31]. However, to the best of our knowledge systematic studies assessing the direct effect of fructose on B- and T-cell function, respectively, in humans are still lacking. Taken together, results of our study suggest that the intake of free fructose in concentrations that are achievable through a ‘normal’ diet can result in an induction in *GLUT5* and *KHK* expression in monocytes in healthy humans. Our results further suggest that at least upon a short-term exposure, similar effects are not found in other blood immune cells e.g., B- und T-cells and that enterocytes metabolize marked amounts of fructose upon exposure. Further studies are needed to determine if a chronic intake of elevated amounts of free fructose also alters fructose uptake and metabolism in monocytes.

Results of previous studies of our group suggested that an increase in dietary free fructose but not in free glucose intake is related with an increased expression of *TLR2* mRNA expression in PBMCs [16]. In line with these findings the challenge of monocytes isolated from buffy coats of healthy donors with fructose was related with an induction of *TLR2* mRNA while *TLR1* and 6 mRNA expression remained unchanged. Similar effects were not found for glucose and in B- or T-cells. The latter finding is somewhat in line with the findings of Jones et al., who found no direct effects of fructose in T-cells [31]. Moreover, the induction of *TLR2* was related with an enhancement of proinflammatory cytokine release when cells were challenged with LTA both when monocytes were directly exposed to the monosaccharide or when being grown beneath Caco-2 cells challenged with fructose. In line with these findings the release of cytokines following the intake of fructose and *ex vivo* stimulation of cells with LTA was also significantly higher than when subjects had consumed maltodextrin (=glucose). It has been shown before that a challenge of monocytes with fructose can alter their metabolic profile and that fructose may alter their phenotype towards a more inflammatory [31] one. Moreover, in the same study, the concomitant feeding of mice with glucose and fructose resulted in increased serum levels of proinflammatory cytokines upon a challenge with lipopolysaccharide and alterations of fructose metabolism [31]. In studies of others the enhanced release of proinflammatory cytokines following the challenge with fructose and Gram-negative bacteria derived endotoxin was primarily related to a fructose-dependent increase in mTORC1 activity [37,38]. In the present study, we found that the induction of *TLR2* expression and subsequent higher responsiveness of monocytes to a challenge with LTA was dependent upon the metabolism of fructose and related decrease in ATP levels as well as induction of SP1. Indeed, the concomitant treatment with a KHK inhibitor or a SP1 inhibitor attenuated the induction of TLR2 and enhanced the release of proinflammatory cytokines of monocytes when being challenged with LTA. It has been shown by others before that fructose metabolism may acutely deplete cellular ATP levels [39]. Moreover, SP1 has been suggested to be one of the transcription factors involved in the regulation of *TLR2* mRNA expression in humans [22]. It further has been shown that fructose may alter activity of protein phosphatase 2 (PP2A) through cellular ATP levels which in turn has been shown to also alter phosphorylation of SP1 [40]. However, our data also suggest that *SP1* mRNA expression is regulated through mechanisms related to fructose metabolism.

Taken together, results of the present study suggest that the ingestion of fructose may enhance LTA-dependent immune responsiveness in healthy humans through a SP1-dependent induction of *TLR2* expression. Results of the present study by no means preclude that fructose may not also affect responsiveness to bacterial or viral challenges by other means; however, our data suggest that in human monocytes the metabolites derived through the metabolism of fructose may alter *SP1* and subsequently *TLR2* mRNA expression. Further studies are needed to assess if similar alterations are also found when fructose is consumed at elevated levels for an extended period of time or if fructose is consumed in different matrices.

Our intervention studies are not without limitations that need to be considered when interpreting the data. A key limitation is that in both intervention studies only young healthy adults were enrolled. Results may be different in children or elderly as well as in overweight or metabolically diseased subjects. This will have to be assessed in future studies. Also, we only assessed the acute effects of fructose *ex vivo*. It remains to be determined if effects alike are found when fructose is consumed regularly and in settings of ‘real’ infections with Gram-positive bacteria. However, due to ethical reasons studies assessing the effects of fructose in the course of infections *in vivo* would need to focus on animal studies. At least in rodents it has been shown that the regulation of TLR2 in immune cells may differ from that in humans [22]. Also, if effects on *TLR2* expression and signaling differ if fructose is consumed in a natural matrix e.g., as part of fruit (juice) or vegetable remains to be determined, too.

## 5 Conclusion

In summary, results of the present study suggest that an acute elevated intake of fructose may enhance the TLR2-dependent response to LTA in blood monocytes in healthy humans thereby bolstering the hypothesis that certain nutritional factors like sugar may alter immune responsiveness of humans. Our results further suggest that this enhancement of the LTA-dependent immune response is mediated through an activation of the transcription factor SP1. Moreover, results of the present study also suggest that herein the intracellular metabolism of fructose is critical. However, if and how signaling pathways shown in the present study affect disease outcomes in setting of Gram-positive infections in humans and if adaptive responses e.g., a regular consumption of fructose or the matrix in which the sugar is consumed alter the effects of fructose on TLR2 signaling remains to be determined.

## CRediT authorship contribution statement

**Raphaela Staltner:** Writing – review & editing, Writing – original draft, Visualization, Investigation, Formal analysis. **Katja Csarmann:** Writing – review & editing, Investigation. **Amelie Geyer:** Writing – review & editing, Investigation. **Anika Nier:** Writing – review & editing, Investigation. **Anja Baumann:** Writing – review & editing, Investigation. **Ina Bergheim:** Writing – review & editing, Writing – original draft, Supervision, Conceptualization.

## Declaration of competing interest

All authors declare no conflict of interest.

## Figures and Tables

**figs1:**
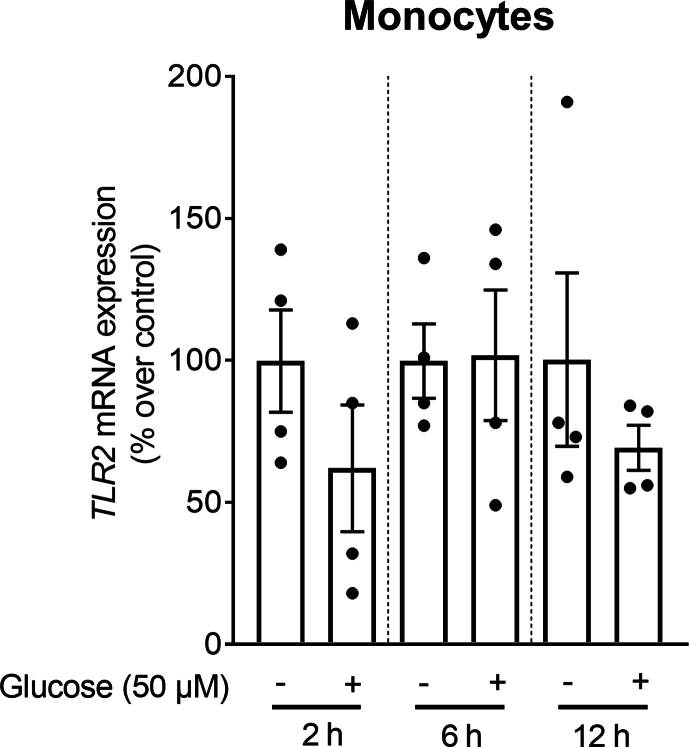


## Data Availability

Data will be made available on request.
