# Supplementary material for: Fructose intake enhances lipoteichoic acid-mediated immune response in monocytes of healthy humans
Source: Redox Biol. 2025 Jun 12;85:103729. doi: 10.1016/j.redox.2025.103729 (PMC12205830; doi:10.1016/j.redox.2025.103729)
Supplement: Multimedia component 1 [file mmc1.docx]

**Fructose intake enhances lipoteichoic acid-mediated immune response in monocytes of healthy humans**

Raphaela Staltner, Katja Csarmann, Amelie Geyer, Anika Nier, Anja Baumann, Ina Bergheim*

Department of Nutritional Sciences, Molecular Nutritional Science, University of Vienna, Vienna, Austria

**Corresponding author*:** Ina Bergheim, Ph.D.

University of Vienna

Department of Nutritional Sciences

Molecular Nutritional Science

Josef-Holaubek-Platz 2 (UZA II)

A-1090 Vienna

Phone: +43-1-4277-549 81

E-Mail: ina.bergheim@univie.ac.at

**Table S1. Characteristics of healthy subjects of study population A.**

| **Parameter** | **Baseline** |
| --- | --- |
| Gender (m/f) | 4/6 |
| Age (years) | 25.3 ± 1.1 |
| BMI (kg/m^2^) | 22.3 ± 0.8 |
| Blood glucose (mg/dL) | 87.8 ± 2.0 |
| Total cholesterol (mg/dL) | 170.7 ± 12.2 |
| Triglycerides (mg/dL) | 72.9 ± 8.6 |
| HDL (mg/dL) | 58.6 ± 4.2 |
| LDL (mg/dL) | 99.0 ± 9.7 |
| Blood pressure |  |
| Systolic blood pressure (mm Hg) | 130.8 ± 3.4 |
| Diastolic blood pressure (mm Hg) | 71.2 ± 4.1 |
| Uric acid (mg/dL) | 5.3 ± 0.4 |
| ALT (U/L) | 31.2 ± 3.7 |
| AST (U/L) | 24.8 ± 2.2 |
| Gamma-GT (U/L) | 15.4 ± 1.4 |

Values are means ± standard error of means. BMI: body mass index; HDL: high density lipoproteins; LDL: low density lipoproteins; ALT: alanine aminotransferase; AST: aspartate aminotransferase; Gamma-GT: gamma glutamyl-transferase.

Table S2. Characteristics of healthy human subjects of study population B.

| **Parameter** | **Baseline** |
| --- | --- |
| Gender (m/f) | 3/4 |
| Age (years) | 29.3 ± 1.0 |
| BMI (kg/m^2^) | 21.2 ± 0.6 |
| Blood glucose (mg/dL) | 81 ± 2 |
| Total cholesterol (mg/dL) | 154 ± 10 |
| Triglycerides (mg/dL) | 70.9 ± 9.0 |
| HDL (mg/dL) | 57.7 ± 4.0 |
| LDL (mg/dL) | 84.3 ± 8.2 |
| Blood pressure |  |
| Systolic blood pressure (mm Hg) | 117.2 ± 2.3 |
| Diastolic blood pressure (mm Hg) | 71.5 ± 3.8 |
| Uric acid (mg/dL) | 4.2 ± 0.5 |
| ALT (U/L) | 22.4 ± 2.0 |
| AST (U/L) | 22.9 ± 4.4 |
| Gamma-GT (U/L) | 12.9 ± 0.9 |

Values are means ± standard error of means. BMI: body mass index; HDL: high density lipoproteins; LDL: low density lipoproteins; ALT: alanine aminotransferase; AST: aspartate aminotransferase; Gamma-GT: gamma glutamyl-transferase.

Table S3. Results of 24-h recalls and nutritional standardization of study population B.

|  | **24-h recall** | **Standardization** |
| --- | --- | --- |
| Energy (kcal/d) | 2204 ± 120 | 2138 ± 75 |
| Carbohydrates (E%) | 42 ± 5 | 59 ± 0* |
| Fiber (g/d) | 29 ± 4 | 33 ± 1 |
| Protein (g/kg body weight/d) | 1.5 ± 0.2 | 0.8 ± 0.0* |
| Fat (E%) | 38 ± 4 | 31 ± 0 |
| SFA (E%) | 13 ± 2 | 9 ± 0* |

Values are means ± standard error of means. E%: percentage of daily energy, **p≤0.05;* SFA: saturated fatty acids.

Table S4. Primer sequences.

|  | **Forward (5´-3´)** | **Reverse (5´-3´)** |
| --- | --- | --- |
| ***18S*** | ttg ccc tcc aat gga tcc tc | acg ggg aat cag ggt tcg at |
| ***GLUT5*** | aag gga ggc tga cgc ttg tg | tga gca gtg ctg ggg agt tg |
| ***KHK*** | cag gtg gcc ggc aag aag tg | ccc cag agc ctc tcc ctg tg |
| ***IL-1β*** | caa ggg ctt cag gca ggc cg | tga gtc ccg gag cgt gca gt |
| ***IL-6*** | cga gcc cac cgg gaa cga aa | gtg gct gtc tgt gtg ggg cg |
| ***SP1*** | aga acc cac aag ccc aaa ca | tgc acc tgg att cct gaa gt |
| ***TLR1*** | agg gtc agc tgg act tca ga | taa ttt tgg atg ggc aaa gc |
| ***TLR2*** | att gtg ccc att gct ctt tc | ctg ccc ttg cag ata cca tt |
| ***TLR6*** | cga tag cca ctg caa cat ca | cag cgg tag gtc ttt tgg aa |
| ***TNF-α*** | aaa aca acc ctc aga cgc cac at | gaa gga gaa gag gct gag gaa caa g |

GLUT5: glucose transporter 5; IL: Interleukin; KHK: ketohexokinase; TLR: Toll-like receptor; TNF-α: Tumor necrosis factor alpha.
